# Crystal structure of *N*-[6-amino-5-(benzo[*d*]thia­zol-2-yl)-3-cyano-4-methyl­sulfanyl-2-oxo-1,2-di­hydro­pyridin-1-yl]-4-methyl­benzene­sulfonamide di­methyl­formamide monosolvate

**DOI:** 10.1107/S2056989017015778

**Published:** 2017-11-03

**Authors:** Rasha A. Azzam, Galal H. Elgemeie, Rasha E. Elsayed, Peter G. Jones

**Affiliations:** a Chemistry Department, Faculty of Science, Helwan University, Cairo, Egypt; b Institut für Anorganische und Analytische Chemie, Technische Universität Braunschweig, Hagenring 30, D-38106 Braunschweig, Germany

**Keywords:** crystal structure, 2-pyridone, benzo­thia­zole, di­methyl­formamide

## Abstract

In the title compound, C_21_H_17_N_5_O_3_S_3_·C_3_H_7_NO, the toluene­sulfonamide ring and the combined ring system involving the pyridone and benzo­thia­zole rings subtend an inter­planar angle of 39.86 (4)°. The pyridone and benzo­thiazyl rings are linked by the intra­molecular hydrogen bond N—H_amine_⋯N_thia­zole_. The DMF O atom accepts two classical hydrogen bonds. The mol­ecules are linked by hydrogen bonds and an S⋯O contact to form layers parallel to the *bc* plane.

## Abstract 2

In the title compound, the toluene­sulfonamide ring and the combined ring system involving the pyridone and benzo­thia­zole rings subtend an inter­planar angle of 39.86 (4)°. The pyridone and benzo­thiazyl rings are linked by an intra­molecular N—H_amine_⋯N_thia­zole_ hydrogen bond. The mol­ecules are linked by hydrogen bonds and an S⋯O contact to form layers parallel to the *bc* plane.

## Chemical context   

Cyano­ketene di­thio­acetals are versatile synthetic inter­mediates (Elgemeie *et al.*, 2003*a* ▸, 2015 ▸) that have been utilized as building blocks for the synthesis of a wide range of heterocyclic compounds (Elgemeie *et al.*, 2009 ▸, 2017*a* ▸); they are also of general inter­est in pharmaceutical chemistry (Elgemeie & Abou-Zeid, 2015 ▸; Elgemeie *et al.*, 2016 ▸). Recently, we have described the synthesis of various anti­metabolites starting from cyano­ketene di­thio­acetals and related compounds, *viz*. cyano­ketene *S*,*S*-acetals (Elgemeie, Mohamed, 2006 ▸), cyano­ketene *N*,*S*-acetals (Elgemeie *et al.* 2017*b* ▸), and cyano­ketene *N*,*N*-acetals (Elgemeie *et al.*, 2003*b* ▸). As a part of this programme, the reaction of 2-(benzo[*d*]thia­zol-2-yl)-3,3-bis­(methyl­thio)­acrylo­nitrile (**1**) with *N*-(2-cyano­acet­yl)-4-methyl­benzene­sulfono­hydrazide (**2**) was investigated. The reaction between **1** and **2** in KOH–DMF gives an adduct for which four possible isomeric structures were considered (structures **3**–**6**). Spectroscopic methods did not allow us to identify the product unambiguously and therefore the X-ray crystal structure was determined, confirming the exclusive presence of structure **6** in the solid state. The formation of **6** from the reaction of **1** and **2** is assumed to proceed *via* initial addition of the active methyl­ene carbon atom of **2** to the double bond of **1**, followed by elimination of CH_3_SH and cyclization *via* addition of the NH group to the cyano group of benzo­thia­zole to give the favoured, kinetically and thermodynamically controlled product **6**. The ^1^H NMR spectra of the product revealed the presence of an amino group at δ = 8.84 p.p.m. and a pyridine methyl­thio group at δ = 2.45 p.p.m. in solution. Compound **6** and its derivatives showed inter­esting preclinical biological results and are currently being patented (Elgemeie *et al.*, 2017*c* ▸).
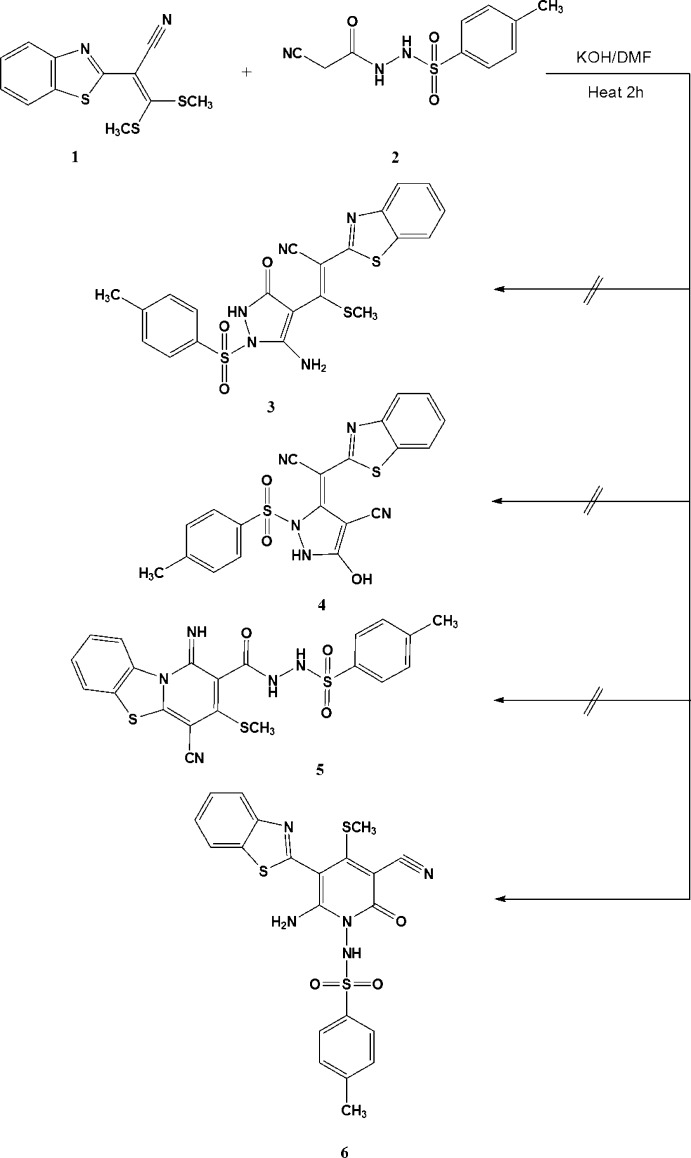


## Structural commentary   

**Figure 1:**
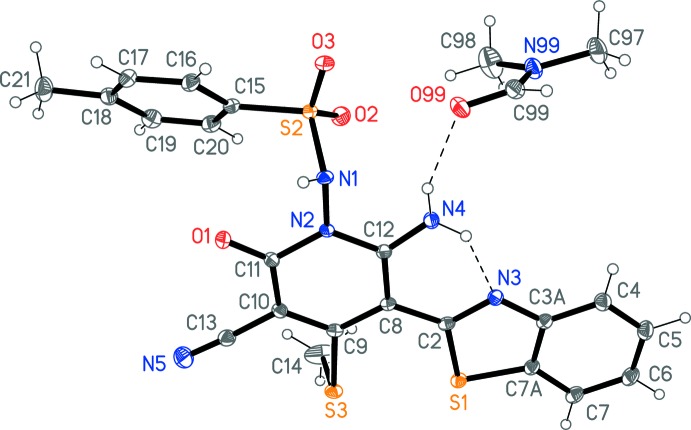
The structure of the title compound in the crystal. Displacement ellipsoids represent 50% probability levels.

The solid-state structure of **6** is shown in Fig. 1 ▸, the structure analysis thereby confirming the nature of the product. The mol­ecule essentially consists of two planes; the toluene­sulfonamide ring and the combined ring system involving the pyridone and benzo­thia­zole rings. The former has a r.m.s. deviation of 0.04 Å and the latter of 0.01 Å (including all direct substituents), and the inter­planar angle is 39.86 (4)°. The pyridone and benzo­thiazyl rings are held coplanar by the intra­molecular hydrogen bond N4—H03⋯N3 (Table 1 ▸). The contact N4—H02⋯N1 might also be classified as a hydrogen bond, with H⋯N 2.24 (2) Å, but its angle is only 105.7 (15)°. The nitro­gen N4 is planar (angle sum 359.7°) but N1 is pyramidalized (343.9°).

## Supra­molecular features   

**Figure 2:**
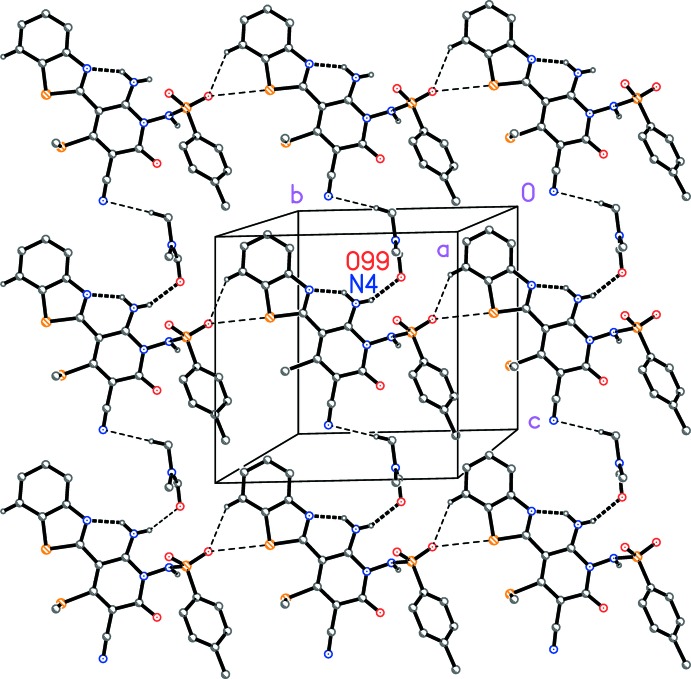
Packing diagram of the title compound viewed perpendicular to the *bc* plane. Dashed lines indicate classical hydrogen bonds (thick) or C—H⋯*X* and S⋯O inter­actions (thin).

The oyxgen atom of the di­methyl­formamide accepts two classical hydrogen bonds. The clearest packing feature is the formation of layers parallel to the *bc* plane (Fig. 2 ▸), in which the hydrogen bonds H02⋯O99, H7⋯O3^ii^ and H97*C*⋯N5^iv^ are involved (Table 1 ▸), together with the short contact S1⋯O3(*x*, 1 + *y*, *z*) 3.2662 (10) Å. The hydrogen bond H01⋯O99^i^ connects the layers in the third dimension.

## Database survey   

The 2-pyridone ring displays the usual features of a narrow angle at nitro­gen and a wide angle at the carbonyl carbon (Table 2 ▸). A database search gave 555 hits (745 values) for the 2-pyridone ring, with average angles of 123.9° at nitro­gen and 115.3° at C=O. No other structures could be found in which a 2-pyridone ring is attached at the 5-position to the C2 atom of a thia­zol ring.

## Synthesis and crystallization   

2-(Benzo[*d*]thia­zol-2-yl)-3,3-bis­(methyl­thio)­acrylo­nitrile (**1**) (2.78 g, 0.01 mol) was added to a solution of *N*-(2-cyano­acet­yl)-4-methyl­benzene­sulfono­hydrazide (**2**) (2.53 g., 0.01 mol) in dry DMF (30 ml) containing pulverized potassium hydrox­ide (0.56 g, 0.01 mol). The reaction mixture was refluxed with stirring for 2 h (TLC monitoring). After cooling, the reaction mixture was poured into ice-cold water and neutralized with HCl. The solid product was filtered off, washed with water, and dried. It was further purified from hot ethyl acetate: petroleum ether (1:1). The precipitated solid was crystallized from DMF to give yellow crystals, m.p. = 494 K, yield 78%.

IR (KBr, cm^−1^): ν 3393, 3208 (NH, NH_2_), 3072 (ArCH), 2922 (CH_3_), 2210 (CN), 1677 (CO), 1594 (C=N), 1350, 1170 (O=S=O); ^1^H NMR (400 MHz, DMSO-*d*_6_): δ 2.42 (*s*, 3H, CH_3_), 2.45 (*s*, 3H, SCH_3_), 7.42 (*d*, *J* = 8 Hz, 2H, C_6_H_4_), 7.49 (*t*, *J* = 8 Hz, 1H, benzo­thia­zole H), 7.56 (*t*, *J* = 8 Hz, 1H, benzo­thia­zole H), 7.71 (*d*, *J* = 8 Hz, 2H, C_6_H_4_), 8.06 (*d*, *J* = 8 Hz, 1H, benzo­thia­zole H), 8.13 (*d*, *J* = 8 Hz, 1H, benzo­thia­zole H), 8.84 (*br*, 2H, NH_2_), 11.44 (*s*, 1H, NH). Analysis calculated for C_21_H_17_N_5_O_3_S_3_ (483.59): C 52.16, H 3.54, N 14.48%; found: C 52.11; H 3.48; N 14.50%; MS *m*/*z* (%): 484 (*M*+1, 1.03%), 384 (84%), 356 (100%), 283 (60%), 117 (77%).

## Refinement   

Crystal data, data collection and structure refinement details are summarized in Table 3 ▸. NH hydrogen atoms were refined freely. Methyl hydrogen atoms were refined as idealized rigid groups allowed to rotate but not tip (AFIX 137), with C—H 0.98 Å and H—C—H 109.5°. Other hydrogen atoms were included using a riding model starting from calculated positions (C—H_aromatic_ 0.95, C—H_methine_ 1.00 Å) with *U*_iso_(H) = 1.5*U*_eq_(C) for methyl H atoms and 1.2*U*_eq_(C) for all others.

## Supplementary Material

Crystal structure: contains datablock(s) I, global. DOI: 10.1107/S2056989017015778/hg5500sup1.cif

Structure factors: contains datablock(s) I. DOI: 10.1107/S2056989017015778/hg5500Isup2.hkl

CCDC reference: 1582798

Additional supporting information:  crystallographic information; 3D view; checkCIF report

## Figures and Tables

**Table 1:** Hydrogen-bond geometry (Å, °)

*D*—H⋯*A*	*D*—H	H⋯*A*	*D*⋯*A*	*D*—H⋯*A*
N1—H01⋯O99^i^	0.888 (18)	1.872 (18)	2.7583 (13)	175.7 (16)
N4—H02⋯O99	0.84 (2)	2.05 (2)	2.8334 (14)	154.6 (18)
N4—H03⋯N3	0.86 (2)	1.86 (2)	2.5760 (15)	139.9 (17)
N4—H02⋯N1	0.84 (2)	2.237 (19)	2.5932 (14)	105.7 (15)
C7—H7⋯O3^ii^	0.95	2.54	3.3161 (16)	139
C20—H20⋯O2^iii^	0.95	2.64	3.5605 (16)	164
C97—H97*C*⋯N5^iv^	0.98	2.59	3.504 (2)	155

Symmetry codes: (i) 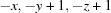; (ii) 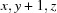; (iii) 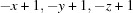; (iv) 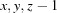.

**Table 2:** Selected bond angles (°)

N2—C11—C10	113.44 (10)	C12—N2—C11	125.63 (10)

**Table 3:** Experimental details

Crystal data	
Chemical formula	C_21_H_17_N_5_O_3_S_3_·C_3_H_7_NO
*M*_r_	556.67
Crystal system, space group	Triclinic, *P* 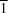
Temperature (K)	100
*a*, *b*, *c* (Å)	9.9916 (5), 11.7805 (6), 11.9776 (6)
α, β, γ (°)	88.809 (4), 79.159 (4), 67.245 (5)
*V* (Å^3^)	1274.80 (12)
*Z*	2
Radiation type	Mo *K*α
μ (mm^−1^)	0.34
Crystal size (mm)	0.5 × 0.4 × 0.2

Data collection	
Diffractometer	Oxford Diffraction Xcalibur Eos
Absorption correction	Multi-scan (*CrysAlis PRO*; Rigaku OD, 2015 ▸)
*T*_min_, *T*_max_	0.972, 1.000
No. of measured, independent and observed [*I* > 2σ(*I*)] reflections	68326, 7630, 6682
*R*_int_	0.036
(sin θ/λ)_max_ (Å^−1^)	0.726

Refinement	
*R*[*F* ^2^ > 2σ(*F* ^2^)], *wR*(*F* ^2^), *S*	0.033, 0.082, 1.04
No. of reflections	7630
No. of parameters	350
H-atom treatment	H atoms treated by a mixture of independent and constrained refinement
Δρ_max_, Δρ_min_ (e Å^−3^)	0.61, −0.36

Computer programs: *CrysAlis PRO* (Rigaku OD, 2015 ▸), *SHELXS97* and *SHELXL97* (Sheldrick, 2008 ▸), *SHELXL2017* (Sheldrick, 2015 ▸) and *XP* (Siemens, 1994 ▸).

## supplementary crystallographic information

### Crystal data

**Table:**

C_21_H_17_N_5_O_3_S_3_·C_3_H_7_NO	*Z* = 2
*M**_r_* = 556.67	*F*(000) = 580
Triclinic, *P*1	*D*_x_ = 1.450 Mg m^−^^3^
*a* = 9.9916 (5) Å	Mo *K*α radiation, λ = 0.71073 Å
*b* = 11.7805 (6) Å	Cell parameters from 19857 reflections
*c* = 11.9776 (6) Å	θ = 2.3–30.6°
α = 88.809 (4)°	µ = 0.34 mm^−^^1^
β = 79.159 (4)°	*T* = 100 K
γ = 67.245 (5)°	Tablet, yellow
*V* = 1274.80 (12) Å^3^	0.5 × 0.4 × 0.2 mm

### Data collection

**Table:**

Oxford Diffraction Xcalibur Eos diffractometer	7630 independent reflections
Radiation source: fine-focus sealed X-ray tube	6682 reflections with *I* > 2σ(*I*)
Detector resolution: 16.1419 pixels mm^-1^	*R*_int_ = 0.036
ω–scan	θ_max_ = 31.1°, θ_min_ = 2.3°
Absorption correction: multi-scan (CrysAlis PRO; Rigaku Oxford Diffraction, 2015)	*h* = −14→14
*T*_min_ = 0.972, *T*_max_ = 1.000	*k* = −16→16
68326 measured reflections	*l* = −17→17

### Refinement

**Table:**

Refinement on *F*^2^	Primary atom site location: structure-invariant direct methods
Least-squares matrix: full	Secondary atom site location: difference Fourier map
*R*[*F*^2^ > 2σ(*F*^2^)] = 0.033	Hydrogen site location: mixed
*wR*(*F*^2^) = 0.082	H atoms treated by a mixture of independent and constrained refinement
*S* = 1.04	*w* = 1/[σ^2^(*F*_o_^2^) + (0.0337*P*)^2^ + 0.772*P*] where *P* = (*F*_o_^2^ + 2*F*_c_^2^)/3
7630 reflections	(Δ/σ)_max_ = 0.001
350 parameters	Δρ_max_ = 0.61 e Å^−^^3^
0 restraints	Δρ_min_ = −0.36 e Å^−^^3^

### Special details

**Table:**

Geometry. All esds (except the esd in the dihedral angle between two l.s. planes) are estimated using the full covariance matrix. The cell esds are taken into account individually in the estimation of esds in distances, angles and torsion angles; correlations between esds in cell parameters are only used when they are defined by crystal symmetry. An approximate (isotropic) treatment of cell esds is used for estimating esds involving l.s. planes.

### Fractional atomic coordinates and isotropic or equivalent isotropic displacement parameters (Å^2^)

**Table:**

	*x*	*y*	*z*	*U*_iso_*/*U*_eq_	
S1	0.20917 (3)	1.05419 (3)	0.44384 (2)	0.01311 (6)	
C2	0.17424 (12)	0.91906 (10)	0.43367 (10)	0.0117 (2)	
N3	0.13892 (11)	0.90316 (9)	0.33646 (8)	0.01298 (18)	
C3A	0.13978 (12)	0.99549 (11)	0.26319 (10)	0.0130 (2)	
C4	0.10933 (14)	1.00118 (12)	0.15352 (10)	0.0165 (2)	
H4	0.086143	0.938809	0.122941	0.020*	
C5	0.11382 (14)	1.10006 (12)	0.09052 (11)	0.0183 (2)	
H5	0.094063	1.105310	0.015622	0.022*	
C6	0.14719 (14)	1.19255 (12)	0.13597 (11)	0.0185 (2)	
H6	0.148137	1.260042	0.091474	0.022*	
C7	0.17885 (14)	1.18784 (11)	0.24436 (11)	0.0167 (2)	
H7	0.201612	1.250566	0.274740	0.020*	
C7A	0.17590 (13)	1.08692 (11)	0.30715 (10)	0.0134 (2)	
C8	0.18455 (12)	0.82947 (10)	0.52233 (10)	0.0113 (2)	
C9	0.21641 (12)	0.84103 (10)	0.63037 (10)	0.0119 (2)	
C10	0.22112 (13)	0.75420 (11)	0.71150 (10)	0.0130 (2)	
C11	0.19296 (13)	0.64662 (11)	0.69151 (10)	0.0126 (2)	
C12	0.16105 (12)	0.72035 (10)	0.49822 (9)	0.0112 (2)	
C13	0.25168 (14)	0.76681 (11)	0.82143 (11)	0.0161 (2)	
C14	0.44732 (16)	0.90452 (15)	0.64337 (18)	0.0375 (4)	
H14A	0.482909	0.832100	0.688175	0.056*	
H14B	0.484276	0.965711	0.663453	0.056*	
H14C	0.483227	0.879848	0.562098	0.056*	
S2	0.28523 (3)	0.39958 (3)	0.51130 (2)	0.01363 (7)	
S3	0.24867 (3)	0.97028 (3)	0.67335 (3)	0.01445 (7)	
O1	0.18556 (10)	0.56948 (8)	0.75942 (7)	0.01650 (17)	
O2	0.39814 (10)	0.43053 (8)	0.44172 (8)	0.01993 (19)	
O3	0.22015 (11)	0.32617 (8)	0.46616 (8)	0.01989 (19)	
N1	0.14142 (11)	0.53246 (9)	0.55209 (8)	0.01215 (18)	
H01	0.0686 (19)	0.5236 (16)	0.6018 (15)	0.024 (4)*	
N2	0.17243 (11)	0.63362 (9)	0.57965 (8)	0.01120 (18)	
N4	0.13014 (12)	0.69607 (10)	0.40197 (9)	0.01497 (19)	
H02	0.116 (2)	0.6318 (18)	0.3914 (16)	0.031 (5)*	
H03	0.118 (2)	0.7536 (18)	0.3545 (16)	0.030 (5)*	
N5	0.27764 (14)	0.77140 (11)	0.91037 (10)	0.0249 (2)	
C15	0.35093 (13)	0.32693 (11)	0.63125 (10)	0.0142 (2)	
C16	0.27838 (14)	0.25780 (11)	0.69169 (11)	0.0162 (2)	
H16	0.196647	0.250018	0.667958	0.019*	
C17	0.32777 (14)	0.20066 (11)	0.78708 (11)	0.0174 (2)	
H17	0.278268	0.154275	0.829314	0.021*	
C18	0.44894 (14)	0.21006 (11)	0.82216 (11)	0.0173 (2)	
C19	0.51825 (14)	0.28051 (12)	0.76017 (11)	0.0184 (2)	
H19	0.600293	0.288172	0.783499	0.022*	
C20	0.46997 (13)	0.33974 (11)	0.66507 (11)	0.0168 (2)	
H20	0.517566	0.388092	0.623997	0.020*	
C21	0.50385 (16)	0.14328 (14)	0.92318 (12)	0.0247 (3)	
H21A	0.419338	0.152364	0.984240	0.037*	
H21B	0.567230	0.178318	0.950152	0.037*	
H21C	0.560779	0.055639	0.901004	0.037*	
C97	0.20835 (19)	0.51005 (15)	−0.00331 (12)	0.0303 (3)	
H97A	0.105803	0.547512	−0.014305	0.045*	
H97B	0.258431	0.429728	−0.045575	0.045*	
H97C	0.259893	0.563981	−0.031259	0.045*	
C98	0.35300 (17)	0.44892 (18)	0.15100 (14)	0.0356 (4)	
H98A	0.339323	0.441322	0.233601	0.053*	
H98B	0.400502	0.507200	0.129860	0.053*	
H98C	0.415656	0.368141	0.112343	0.053*	
C99	0.08612 (15)	0.51648 (12)	0.19300 (11)	0.0186 (2)	
H99	−0.003677	0.541604	0.165524	0.022*	
N99	0.20970 (13)	0.49363 (11)	0.11715 (9)	0.0198 (2)	
O99	0.07862 (10)	0.50763 (9)	0.29665 (7)	0.01853 (18)	

### Atomic displacement parameters (Å^2^)

**Table:**

	*U*^11^	*U*^22^	*U*^33^	*U*^12^	*U*^13^	*U*^23^
S1	0.01528 (13)	0.01090 (13)	0.01511 (13)	−0.00706 (10)	−0.00348 (10)	0.00176 (10)
C2	0.0104 (5)	0.0097 (5)	0.0145 (5)	−0.0042 (4)	−0.0008 (4)	0.0002 (4)
N3	0.0147 (4)	0.0121 (4)	0.0126 (4)	−0.0060 (4)	−0.0022 (3)	0.0015 (3)
C3A	0.0114 (5)	0.0121 (5)	0.0137 (5)	−0.0038 (4)	−0.0004 (4)	0.0013 (4)
C4	0.0173 (5)	0.0168 (5)	0.0156 (5)	−0.0069 (4)	−0.0032 (4)	0.0016 (4)
C5	0.0184 (6)	0.0200 (6)	0.0148 (5)	−0.0059 (5)	−0.0029 (4)	0.0046 (4)
C6	0.0188 (6)	0.0156 (6)	0.0195 (6)	−0.0062 (5)	−0.0020 (5)	0.0059 (4)
C7	0.0174 (5)	0.0130 (5)	0.0198 (6)	−0.0067 (4)	−0.0021 (4)	0.0038 (4)
C7A	0.0124 (5)	0.0122 (5)	0.0143 (5)	−0.0042 (4)	−0.0013 (4)	0.0020 (4)
C8	0.0114 (5)	0.0092 (5)	0.0130 (5)	−0.0041 (4)	−0.0016 (4)	−0.0003 (4)
C9	0.0108 (5)	0.0108 (5)	0.0139 (5)	−0.0041 (4)	−0.0017 (4)	−0.0015 (4)
C10	0.0140 (5)	0.0127 (5)	0.0123 (5)	−0.0049 (4)	−0.0032 (4)	−0.0008 (4)
C11	0.0133 (5)	0.0126 (5)	0.0108 (5)	−0.0039 (4)	−0.0023 (4)	−0.0005 (4)
C12	0.0115 (5)	0.0105 (5)	0.0113 (5)	−0.0044 (4)	−0.0007 (4)	0.0004 (4)
C13	0.0178 (5)	0.0139 (5)	0.0172 (6)	−0.0060 (4)	−0.0053 (4)	0.0001 (4)
C14	0.0149 (6)	0.0276 (8)	0.0685 (12)	−0.0089 (6)	−0.0016 (7)	−0.0143 (8)
S2	0.01854 (14)	0.00982 (12)	0.01155 (13)	−0.00509 (10)	−0.00151 (10)	−0.00011 (9)
S3	0.01584 (13)	0.01206 (13)	0.01707 (14)	−0.00662 (10)	−0.00418 (10)	−0.00170 (10)
O1	0.0234 (4)	0.0140 (4)	0.0126 (4)	−0.0076 (3)	−0.0040 (3)	0.0024 (3)
O2	0.0211 (4)	0.0179 (4)	0.0164 (4)	−0.0061 (4)	0.0033 (3)	0.0016 (3)
O3	0.0315 (5)	0.0121 (4)	0.0181 (4)	−0.0088 (4)	−0.0089 (4)	−0.0005 (3)
N1	0.0155 (5)	0.0092 (4)	0.0131 (4)	−0.0067 (4)	−0.0018 (4)	0.0001 (3)
N2	0.0153 (4)	0.0090 (4)	0.0108 (4)	−0.0063 (3)	−0.0028 (3)	0.0000 (3)
N4	0.0237 (5)	0.0127 (5)	0.0129 (5)	−0.0107 (4)	−0.0062 (4)	0.0025 (4)
N5	0.0317 (6)	0.0252 (6)	0.0212 (6)	−0.0117 (5)	−0.0121 (5)	0.0009 (5)
C15	0.0168 (5)	0.0100 (5)	0.0139 (5)	−0.0038 (4)	−0.0017 (4)	0.0002 (4)
C16	0.0190 (6)	0.0137 (5)	0.0177 (6)	−0.0080 (4)	−0.0046 (4)	0.0019 (4)
C17	0.0210 (6)	0.0139 (5)	0.0172 (6)	−0.0073 (5)	−0.0030 (4)	0.0025 (4)
C18	0.0171 (5)	0.0147 (5)	0.0152 (5)	−0.0013 (4)	−0.0026 (4)	−0.0005 (4)
C19	0.0130 (5)	0.0194 (6)	0.0208 (6)	−0.0040 (4)	−0.0030 (4)	−0.0009 (5)
C20	0.0145 (5)	0.0151 (5)	0.0190 (6)	−0.0054 (4)	0.0001 (4)	−0.0003 (4)
C21	0.0214 (6)	0.0284 (7)	0.0198 (6)	−0.0041 (5)	−0.0063 (5)	0.0065 (5)
C97	0.0417 (9)	0.0361 (8)	0.0130 (6)	−0.0163 (7)	−0.0027 (6)	0.0032 (5)
C98	0.0233 (7)	0.0561 (11)	0.0247 (7)	−0.0136 (7)	−0.0018 (6)	−0.0001 (7)
C99	0.0228 (6)	0.0182 (6)	0.0164 (6)	−0.0094 (5)	−0.0045 (5)	0.0006 (4)
N99	0.0238 (5)	0.0229 (5)	0.0126 (5)	−0.0098 (4)	−0.0021 (4)	0.0011 (4)
O99	0.0248 (5)	0.0228 (5)	0.0127 (4)	−0.0153 (4)	−0.0016 (3)	−0.0004 (3)

### Geometric parameters (Å, º)

**Table:**

S1—C7A	1.7375 (12)	S2—N1	1.6678 (10)
S1—C2	1.7677 (12)	S2—C15	1.7597 (12)
C2—N3	1.3153 (15)	N1—N2	1.4020 (13)
C2—C8	1.4706 (15)	N1—H01	0.888 (18)
N3—C3A	1.3848 (14)	N4—H02	0.84 (2)
C3A—C4	1.3977 (17)	N4—H03	0.86 (2)
C3A—C7A	1.4013 (17)	C15—C20	1.3872 (17)
C4—C5	1.3855 (17)	C15—C16	1.3955 (17)
C4—H4	0.9500	C16—C17	1.3875 (17)
C5—C6	1.4031 (19)	C16—H16	0.9500
C5—H5	0.9500	C17—C18	1.3970 (18)
C6—C7	1.3880 (18)	C17—H17	0.9500
C6—H6	0.9500	C18—C19	1.3948 (18)
C7—C7A	1.4005 (16)	C18—C21	1.5034 (18)
C7—H7	0.9500	C19—C20	1.3894 (18)
C8—C9	1.4108 (16)	C19—H19	0.9500
C8—C12	1.4372 (15)	C20—H20	0.9500
C9—C10	1.3897 (16)	C21—H21A	0.9800
C9—S3	1.7781 (12)	C21—H21B	0.9800
C10—C13	1.4295 (16)	C21—H21C	0.9800
C10—C11	1.4340 (16)	C97—N99	1.4536 (17)
C11—O1	1.2213 (14)	C97—H97A	0.9800
C11—N2	1.4132 (14)	C97—H97B	0.9800
C12—N4	1.3124 (15)	C97—H97C	0.9800
C12—N2	1.3851 (14)	C98—N99	1.4554 (19)
C13—N5	1.1499 (17)	C98—H98A	0.9800
C14—S3	1.7952 (15)	C98—H98B	0.9800
C14—H14A	0.9800	C98—H98C	0.9800
C14—H14B	0.9800	C99—O99	1.2343 (15)
C14—H14C	0.9800	C99—N99	1.3242 (17)
S2—O3	1.4317 (10)	C99—H99	0.9500
S2—O2	1.4326 (9)		
			
C7A—S1—C2	89.58 (6)	N2—N1—S2	117.20 (8)
N3—C2—C8	121.49 (10)	N2—N1—H01	113.6 (11)
N3—C2—S1	113.55 (8)	S2—N1—H01	113.1 (11)
C8—C2—S1	124.95 (9)	C12—N2—N1	115.94 (9)
C2—N3—C3A	112.58 (10)	C12—N2—C11	125.63 (10)
N3—C3A—C4	125.08 (11)	N1—N2—C11	117.88 (9)
N3—C3A—C7A	114.40 (10)	C12—N4—H02	121.2 (13)
C4—C3A—C7A	120.52 (11)	C12—N4—H03	114.9 (13)
C5—C4—C3A	118.33 (12)	H02—N4—H03	123.6 (18)
C5—C4—H4	120.8	C20—C15—C16	121.33 (11)
C3A—C4—H4	120.8	C20—C15—S2	120.72 (9)
C4—C5—C6	120.82 (12)	C16—C15—S2	117.94 (9)
C4—C5—H5	119.6	C17—C16—C15	118.80 (12)
C6—C5—H5	119.6	C17—C16—H16	120.6
C7—C6—C5	121.60 (11)	C15—C16—H16	120.6
C7—C6—H6	119.2	C16—C17—C18	121.22 (12)
C5—C6—H6	119.2	C16—C17—H17	119.4
C6—C7—C7A	117.34 (12)	C18—C17—H17	119.4
C6—C7—H7	121.3	C19—C18—C17	118.44 (11)
C7A—C7—H7	121.3	C19—C18—C21	121.38 (12)
C7—C7A—C3A	121.37 (11)	C17—C18—C21	120.17 (12)
C7—C7A—S1	128.74 (10)	C20—C19—C18	121.46 (12)
C3A—C7A—S1	109.88 (8)	C20—C19—H19	119.3
C9—C8—C12	116.48 (10)	C18—C19—H19	119.3
C9—C8—C2	125.41 (10)	C15—C20—C19	118.73 (11)
C12—C8—C2	118.11 (10)	C15—C20—H20	120.6
C10—C9—C8	122.53 (10)	C19—C20—H20	120.6
C10—C9—S3	115.37 (9)	C18—C21—H21A	109.5
C8—C9—S3	122.08 (9)	C18—C21—H21B	109.5
C9—C10—C13	122.46 (11)	H21A—C21—H21B	109.5
C9—C10—C11	122.30 (10)	C18—C21—H21C	109.5
C13—C10—C11	115.24 (10)	H21A—C21—H21C	109.5
O1—C11—N2	119.46 (11)	H21B—C21—H21C	109.5
O1—C11—C10	127.10 (11)	N99—C97—H97A	109.5
N2—C11—C10	113.44 (10)	N99—C97—H97B	109.5
N4—C12—N2	116.83 (10)	H97A—C97—H97B	109.5
N4—C12—C8	123.94 (11)	N99—C97—H97C	109.5
N2—C12—C8	119.23 (10)	H97A—C97—H97C	109.5
N5—C13—C10	176.92 (13)	H97B—C97—H97C	109.5
S3—C14—H14A	109.5	N99—C98—H98A	109.5
S3—C14—H14B	109.5	N99—C98—H98B	109.5
H14A—C14—H14B	109.5	H98A—C98—H98B	109.5
S3—C14—H14C	109.5	N99—C98—H98C	109.5
H14A—C14—H14C	109.5	H98A—C98—H98C	109.5
H14B—C14—H14C	109.5	H98B—C98—H98C	109.5
O3—S2—O2	121.42 (6)	O99—C99—N99	125.03 (13)
O3—S2—N1	102.99 (5)	O99—C99—H99	117.5
O2—S2—N1	106.32 (5)	N99—C99—H99	117.5
O3—S2—C15	106.76 (6)	C99—N99—C97	121.56 (12)
O2—S2—C15	109.03 (6)	C99—N99—C98	121.18 (12)
N1—S2—C15	109.88 (5)	C97—N99—C98	117.25 (12)
C9—S3—C14	98.98 (6)		
			
C7A—S1—C2—N3	−0.89 (9)	C2—C8—C12—N4	−0.37 (17)
C7A—S1—C2—C8	178.19 (10)	C9—C8—C12—N2	−0.79 (15)
C8—C2—N3—C3A	−178.06 (10)	C2—C8—C12—N2	179.01 (10)
S1—C2—N3—C3A	1.06 (13)	C10—C9—S3—C14	83.40 (11)
C2—N3—C3A—C4	178.78 (11)	C8—C9—S3—C14	−98.15 (12)
C2—N3—C3A—C7A	−0.70 (14)	O3—S2—N1—N2	−167.59 (8)
N3—C3A—C4—C5	179.67 (11)	O2—S2—N1—N2	−38.90 (9)
C7A—C3A—C4—C5	−0.89 (17)	C15—S2—N1—N2	78.95 (9)
C3A—C4—C5—C6	−0.39 (18)	N4—C12—N2—N1	−3.39 (15)
C4—C5—C6—C7	0.93 (19)	C8—C12—N2—N1	177.19 (10)
C5—C6—C7—C7A	−0.15 (18)	N4—C12—N2—C11	−174.61 (10)
C6—C7—C7A—C3A	−1.15 (17)	C8—C12—N2—C11	5.97 (17)
C6—C7—C7A—S1	−179.72 (9)	S2—N1—N2—C12	103.12 (10)
N3—C3A—C7A—C7	−178.80 (11)	S2—N1—N2—C11	−84.95 (11)
C4—C3A—C7A—C7	1.70 (17)	O1—C11—N2—C12	172.21 (11)
N3—C3A—C7A—S1	0.01 (13)	C10—C11—N2—C12	−7.76 (16)
C4—C3A—C7A—S1	−179.49 (9)	O1—C11—N2—N1	1.15 (16)
C2—S1—C7A—C7	179.17 (12)	C10—C11—N2—N1	−178.82 (9)
C2—S1—C7A—C3A	0.47 (9)	O3—S2—C15—C20	152.17 (10)
N3—C2—C8—C9	−177.62 (11)	O2—S2—C15—C20	19.34 (12)
S1—C2—C8—C9	3.36 (16)	N1—S2—C15—C20	−96.82 (10)
N3—C2—C8—C12	2.61 (16)	O3—S2—C15—C16	−28.68 (11)
S1—C2—C8—C12	−176.41 (8)	O2—S2—C15—C16	−161.51 (9)
C12—C8—C9—C10	−1.79 (16)	N1—S2—C15—C16	82.33 (10)
C2—C8—C9—C10	178.44 (11)	C20—C15—C16—C17	−0.33 (18)
C12—C8—C9—S3	179.88 (8)	S2—C15—C16—C17	−179.47 (9)
C2—C8—C9—S3	0.10 (16)	C15—C16—C17—C18	−0.79 (19)
C8—C9—C10—C13	−179.23 (11)	C16—C17—C18—C19	1.19 (18)
S3—C9—C10—C13	−0.79 (15)	C16—C17—C18—C21	−177.66 (12)
C8—C9—C10—C11	−0.40 (18)	C17—C18—C19—C20	−0.49 (19)
S3—C9—C10—C11	178.04 (9)	C21—C18—C19—C20	178.35 (12)
C9—C10—C11—O1	−175.15 (12)	C16—C15—C20—C19	1.01 (18)
C13—C10—C11—O1	3.76 (18)	S2—C15—C20—C19	−179.87 (9)
C9—C10—C11—N2	4.82 (16)	C18—C19—C20—C15	−0.59 (19)
C13—C10—C11—N2	−176.27 (10)	O99—C99—N99—C97	−178.03 (13)
C9—C8—C12—N4	179.84 (11)	O99—C99—N99—C98	3.0 (2)

### Hydrogen-bond geometry (Å, º)

**Table:**

*D*—H···*A*	*D*—H	H···*A*	*D*···*A*	*D*—H···*A*
N1—H01···O99^i^	0.888 (18)	1.872 (18)	2.7583 (13)	175.7 (16)
N4—H02···O99	0.84 (2)	2.05 (2)	2.8334 (14)	154.6 (18)
N4—H03···N3	0.86 (2)	1.86 (2)	2.5760 (15)	139.9 (17)
N4—H02···N1	0.84 (2)	2.237 (19)	2.5932 (14)	105.7 (15)
C7—H7···O3^ii^	0.95	2.54	3.3161 (16)	139
C20—H20···O2^iii^	0.95	2.64	3.5605 (16)	164
C97—H97*C*···N5^iv^	0.98	2.59	3.504 (2)	155

Symmetry codes: (i) −*x*, −*y*+1, −*z*+1; (ii) *x*, *y*+1, *z*; (iii) −*x*+1, −*y*+1, −*z*+1; (iv) *x*, *y*, *z*−1.
